# Effectiveness of various dosage parameters of Wii Fit Plus exergames for enhancing balance and postural control among people post-stroke: a systematic review protocol

**DOI:** 10.11124/JBIES-25-00112

**Published:** 2026-01-13

**Authors:** Sayan Pratihar, Karthiga Rajasekaran, Balamurugan Janakiraman

**Affiliations:** SRM College of Physiotherapy, Faculty of Medicine and Health Sciences, SRM Institute of Science and Technology (SRM IST), Kattankulathur, Tamil Nadu, India

**Keywords:** balance, dosage parameters, postural control, stroke, Wii Fit Plus

## Abstract

**Objective:**

The objective of this review is to synthesize the evidence on the effectiveness of various dosage parameters (session duration, frequency, and total intervention period), including their potential dose-response impact (if meta-analysis is feasible), on postural control and balance using Wii Fit Plus balance exergaming in patients post-stroke.

**Introduction:**

Wii Fit Plus is an innovative exergaming tool that consists of interactive standing balance exercises to improve balance and postural control in patients with stroke. However, the optimal treatment effects and dosage response for achieving these outcomes remain unclear.

**Eligibility criteria:**

This review will consider randomized controlled trials, quasi-experimental studies, and other types of experimental studies evaluating preliminary efficacy or effectiveness of Wii Fit Plus balance exergames for adults and older adults post-stroke. The comparators will include standard-care physiotherapy and alternative exergaming interventions. The outcomes of interest will be postural control and balance, quality of life, functional independence, gait performance, and functional mobility.

**Methods:**

A systematic search will be conducted across PubMed, Embase, Scopus, PEDro, Web of Science, and CINAHL (EBSCOhost) for studies published from 2010 onward. The review will follow the JBI methodology for systematic reviews of effectiveness. Study selection, data extraction, and critical appraisal will be independently performed by 2 reviewers. Dose-response meta-analysis using a random effects model will be used to estimate pooled effect size, with subgroup analyses exploring dosing variations. If meta-analysis is not feasible, a narrative synthesis will be conducted. The overall strength of the evidence will be determined using the Grading of Recommendations, Assessment, Development and Evaluation (GRADE) approach.

**Review registration:**

PROSPERO CRD42025641558

## Introduction

The life-threatening consequences of stroke often lead to balance and postural control impairments, affecting approximately 51.5% of stroke survivors. These capabilities are critical for maintaining functional independence and overall quality of life.^1^,^2^ Impaired balance and loss of postural control not only interrupt the activities of daily living but also increase the chances of falls, resulting in injuries among patients with stroke.^3^ The socioeconomic burden is exacerbated by the need for long-term care and rehabilitation, which can be financially challenging for the caregivers of stroke survivors.^4^ Effective treatment strategies are crucial for targeting balance and postural control to improve outcomes and facilitate recovery. Standard physiotherapy interventions in stroke rehabilitation have shown varying degrees of success; however, such treatment approaches are unable to address the specific needs of individual stroke patients adequately due to a lack of personalization, which decreases the likelihood of patients attending therapy sessions.^5^ Such limitations have prompted research into alternative treatment approaches such as exergames, which combine physical exercise with interactive video games, providing both therapeutic benefits and an engaging experience.^6^

Among these innovative interventions, Wii Fit Plus exergames have emerged as a promising tool for neurological rehabilitation because of their engaging and interactive exercises specifically aimed at improving balance and postural control.^7^ The Wii Fit intervention is an effective addition to conventional rehabilitation techniques for stroke survivors, considering its affordability, accessibility, and ability to provide task-specific and repetitive training.^8^

However, the effects of different dosage parameters, such as session duration, frequency, and total intervention period, of Wii Fit Plus exergames on balance and gait performance among stroke patients remain unclear.^9^ A study reported that a 60-minute training session with Wii Fit Plus effectively improved gait and walking performance in chronic stroke patients,^10^ but there are also several studies that conclude that stroke patients could benefit from shorter training sessions (duration ≤30 minutes) for reduced spasticity, enhancing gait and functional activities of daily living.^11^-^13^ Wii Fit Plus training sessions varied in frequency too; for example, 3 sessions per week were initiated in several studies and significantly improved balance and gait.^7^,^10^ Other studies have reported frequencies of 2 and 5 sessions per week, which also demonstrated improved dynamic stability and standing balance in patients with stroke.^14^,^15^ While some studies reported greater improvements in functional outcomes with longer intervention periods, others demonstrated meaningful gains with shorter periods of Wii Fit training.^16^,^17^ This variation reflects the current uncertainty surrounding the optimal dose-response relationship when using Wii Fit Plus in stroke rehabilitation.

Despite the fact that numerous randomized controlled trials have investigated the effectiveness of Wii Fit Plus exergames on balance and postural control in stroke rehabilitation, there is currently no comprehensive systematic review synthesizing the impact of dosage parameters, including their potential dose response relationship in Wii Fit Plus interventions.^18^,^19^ Previous reviews have reported the effects of exergaming and game control features.^20^,^21^ One narrative review reported conflicting dosages and parameters in Wii Fit exergaming, emphasizing the need for consensus on an effective dose.^22^ A preliminary search of PROSPERO, PubMed, the Cochrane Database of Systematic Reviews, and *JBI Evidence Synthesis* was conducted in June 2025, and found no recent or in-progress systematic reviews on the topic. Hence, the findings of this review will yield valuable insights for rehabilitation professionals, clinicians, and researchers to optimize intervention strategies. Furthermore, the review will also contribute to the evidence-based clinical decision-making process, which will ultimately improve rehabilitation outcomes and direct future research on the customized use of exergames for stroke recovery.

The objective of this review is to synthesize the evidence on the effectiveness of various dosage parameters (ie, session duration, frequency, and total intervention period) and analyze the potential dose-response impact (if meta-analysis is feasible) on balance and postural control outcomes from using Wii Fit Plus balance exergames administered in clinical settings for individuals recovering from stroke.

## Review question

What are the effects of various dosage parameters (session duration, frequency, and total intervention period) of Wii Fit Plus exergames in clinical settings on postural control and balance outcomes among patients with stroke aged 18 years or older, compared to standard-care physiotherapy or alternative exergame interventions?

## Eligibility criteria

The PICO approach was employed to guide the eligibility criteria for this review.

### Participants

This systematic review will include studies involving inpatient or outpatient adults (aged 18–64 years) and older adults (aged 65 years or older) diagnosed with ischemic or hemorrhagic stroke confirmed by clinical assessment/neuroimaging. Patients with stroke at any stage of recovery (acute, subacute, or chronic) and with any level of functional ability will be included, provided they have sufficient cognitive function to engage in Wii Fit Plus exergame training and outcome assessments, as defined by the original study authors. Studies involving participants with transient ischemic attack, age <18 years, moderate to severe cognitive impairment, neurodegenerative disorders, or co-existing conditions that may independently affect balance will be excluded unless relevant stroke-specific data can be extracted separately.

### Intervention

This review will include studies reporting on Wii Fit Plus balance-focused exergame training for postural balance improvements in clinical settings. Only standing Wii Fit Plus balance games (eg, Table Tilt, Ski Slalom, Balance Bubble, Penguin Slide, Soccer Heading, Ski Jump, Tightrope Walk, Snowboard Slalom) will be considered. Studies must report specific dosage parameters (session duration, frequency, and total intervention period). Studies using non-balance games within Wii Fit Plus programs (eg, aerobic or strength-focused) or lacking dosage details will be excluded. Studies where participants used assistance or played exergames unassisted will be included, provided they meet all other eligibility criteria.

### Comparators

This review will include studies with either passive or active comparators. Passive comparators may include standard-care physiotherapy. Active comparators may involve alternative interventions, such as other immersive or non-immersive virtual reality–based exergame training programs. Studies without a comparator or control group will be excluded.

### Outcomes

Though no relevant core outcome set was found in the COMET database, outcomes were selected based on clinical relevance and the exisiting literature. The primary outcomes of interest will be postural control and balance, which are key therapeutic targets of Wii Fit Plus balance exergame interventions. The following validated tools will be used in this review: the Balance Evaluation System Test (BESTest), Berg Balance Scale (BBS), Functional Reach Test (FRT), Short Physical Performance Battery (SPPB), centre of pressure sway measures, and other reported balance metrics. Although these are considered surrogate outcomes, they are widely accepted as clinically meaningful indicators of fall risk and functional stability in stroke rehabilitation.^23^-^27^

Secondary outcomes will include quality of life, functional independence, gait performance, and functional mobility, assessed using tools such as the Stroke Impact Scale (SIS), Timed Up and Go (TUG) test, Dynamic Gait Index (DGI), 10-m walk test, Stroke-Specific Quality of Life (SS-QOL), Functional Independence Measures (FIMs), and EQ-5D. These will provide additional insights into the broader impact of the exergames on stroke rehabilitation, helping to understand their clinical relevance beyond balance improvements. Any adverse effects or harms associated with the intervention, such as dizziness, fatigue, falls during training, or dropout due to intolerance, will also be extracted if reported. Studies without outcome assessments or non-clinical feasibility studies will be excluded.

### Types of studies

This systematic review will encompass randomized controlled trials, quasi-experimental studies, and other experimental study designs, including pre-post intervention studies. These studies must evaluate the preliminary efficacy or effect of Wii Fit Plus training as either the sole intervention or as part of stroke rehabilitation, and report measurable clinical outcomes. This systematic review will not consider observational studies (eg, cohort studies, case control studies, cross-sectional studies, case reports, case series, conference abstracts, or non-peer-reviewed gray literature) due to their inability to establish causal relationships and meet the review objective focused on treatment efficacy and dosage-response synthesis.

Studies involving mixed populations (eg, combined neurological conditions) or mixed interventions (eg, different exergame platforms) will be included only if data specific to adult or older adult patients with ischemic or hemorrhagic stroke undergoing Wii Fit Plus balance training are reported separately. The data must include dosage parameters and relevant outcomes as defined in the PICO. If the data for each subgroup cannot be clearly extracted, the study will be excluded. The review will strictly adhere to the predefined eligibility criteria and, in line with JBI guidelines, a list of excluded studies (with reasons) will be presented in the final review.

## Methods

This systematic review will be conducted in accordance with JBI methodology for systematic reviews of effectiveness^28^ and will be reported following the Preferred Reporting Items for Systematic Reviews and Meta-Analyses (PRISMA) checklist.^29^ The protocol has been registered in PROSPERO (CRD42025641558).

### Search strategy

The search strategy will follow the 3-phase process recommended by the *JBI Manual for Evidence Synthesis*. In the first phase, an initial limited search of PubMed and CINAHL will be conducted to identify articles on the topic by 1 reviewer (SP). Text words from titles, abstracts, and index terms of retrieved papers will be analyzed to refine the search terms. In the second phase, the finalized keywords and index terms will be used to construct tailored search strategies for PubMed, Embase, Scopus, Web of Science, PEDro, and CINAHL (EBSCOhost) by the same reviewer in consultation with an experienced librarian. In the third phase, the reference lists and bibliographies of all included studies will be hand-searched by 2 independent reviewers (SP and KR) to identify additional eligible articles. In addition to published studies, gray literature will also be considered, and we will search Google Scholar, dissertation databases, and trial registries such as ClinicalTrials.gov and the WHO International Clinical Trials Registry Platform (ICTRP). Two reviewers (SP and KR) will independently screen the search results for eligibility at the title/abstract and full-text levels. Any disagreements will be resolved through discussion or with a third reviewer (BJ). This dual-screening process will help minimize the impact of reviewer preference (selection bias), ensure a reliable search process, and improve the chances of identifying all relevant articles.^30^

There will be no language restrictions in the search. Articles written in languages other than English will be translated using Google Translate. If they are deemed suitable for inclusion, the full text will then be translated and verified by professional translators to ensure accuracy. The search will be conducted from January 2010 onward, as Wii Fit Plus was introduced in 2009, and we expect to find the earliest related clinical research from 2010. A detailed PubMed search strategy is provided in Appendix I. This strategy will be adapted for each database using controlled vocabulary (eg, MeSH for PubMed, Emtree for Embase) and free-text keywords, in combination with Boolean operators and truncations.

### Study selection

Following the search, all identified citations will be collated and uploaded into Zotero (Corporation for Digital Scholarship and Roy Rosenzweig Center for History and New Media, VA, USA), and duplicates removed. A pilot test will be conducted to ensure consistency in applying the eligibility criteria. A random subset of studies (n=5 or 50% of eligible studies, whichever is greater) will be independently screened by the 2 reviewers. Discrepancies will be discussed and resolved to refine the approach before the full screening begins. Titles and abstracts of all retrieved studies will be screened independently by 2 reviewers (SP and KR) against the eligibility criteria. The full texts of potentially relevant studies will be retrieved separately via institutional access or by contacting study authors, where required, and assessed for eligibility. If any of the review authors’ own studies are identified as potentially eligible, that reviewer will abstain from the selection and critical appraisal to mitigate potential conflicts of interest. Potentially relevant articles will be uploaded into Zotero, and full-text articles will be imported into the JBI System for the Unified Management, Assessment and Review of Information (JBI SUMARI; JBI, Adelaide, Australia).^31^ Any disagreements between reviewers at any stage of the study selection process will be resolved through discussion. If consensus cannot be reached, the third reviewer (BJ) will be consulted. The results of the search and selection process will be reported in a PRISMA flow diagram,^29^ including the number of studies screened, assessed for eligibility, and included in the review, with reasons for exclusion at each stage.

### Assessment of methodological quality

All eligible studies will be critically appraised for methodological quality using the appropriate standardized JBI critical appraisal checklists for randomized controlled trials and quasi-experimental studies.^32^,^33^ Two independent reviewers (SP and KR) will conduct the appraisal at the study and outcome levels, and any disagreements will be resolved through discussion or in consultation with the third reviewer (BJ). Prior to full critical appraisal, a pilot test will be conducted on a small subset of studies (n=3 or 30% of total eligible studies, whichever is higher) to ensure consistency between reviewers in applying the JBI critical appraisal tools. Any discrepancies will be discussed to refine the appraisal process before proceeding with the full review. The results of the critical appraisal will be presented in a summary table. No arbitrary numerical thresholds will be used to exclude studies; instead, the risk of bias judgments (low, moderate, high) across domains will be used to inform the interpretation of findings and guide subgroup and sensitivity analyses. Selective outcome reporting will be assessed by comparing reported outcomes with trial protocols or registry data, when available. Inconsistencies or missing outcome data will be documented and discussed in the synthesis. The critical appraisal findings will directly inform the confidence in the review findings and will be integrated with GRADE assessments where applicable.

### Data extraction

Data will be extracted from the included studies by 2 independent reviewers (SP and KR) using a data extraction form, which was initially developed by the research team in Microsoft Excel (Microsoft, Washington, USA). The following information will be extracted from each included study: study identification, study design, setting, participants, intervention, comparator, outcomes measured, results, adverse effects, and risk of bias notes (see Appendix II). Prior to full data extraction, a pilot test will be conducted on a subset of studies (n=3 or 30% of total eligible studies, whichever is greater) to assess consistency between the reviewers and refine the tool as needed. If key data (eg, dosage details or outcome measures) are missing or unclear, the study authors will be contacted via email up to 2 times for clarification. If no response is received, the data will be recorded as not available. Any disagreements between reviewers will be resolved through discussion. If consensus is not achieved, the third reviewer (BJ) will adjudicate. Inter-rater reliability during data extraction will be assessed using Cohen’s κ.^34^

### Data synthesis

Studies will be pooled using random effects meta-analysis in JBI SUMARI. For additional or advanced analyses, including dose-response meta-analysis, to estimate the relationship between the dosage parameters and treatment outcomes, and multilevel modeling, RevMan v.5 (The Nordic Cochrane Centre, Copenhagen, Denmark) and R software (metafor and dosresmeta packages; R Foundation for Statistical Computing, Vienna, Austria) will be used, where possible. Continuous outcomes will be synthesized using mean differences or standardized mean differences, with 95% CI. Dichotomous outcomes, if applicable, will be analyzed using risk ratios or odds ratios, with 95% CI. Statistical heterogeneity will be assessed using the χ^2^ test (*p* < 0.10) and the *I^2^* statistic, interpreted as low (25%), moderate (50%), or high (75%) heterogeneity. Additional measures, such as Kendall’s τ, may be used, if required. If sufficient data are available, subgroup analyses will be conducted based on stroke type (ischemic vs. hemorrhagic), age (18–65 vs. >65 years), stroke chronicity (≤6 months vs. >6 months), session duration (≤30 minutes vs. >30 minutes), frequency (2–3 vs. >3 sessions/week), and total intervention period (≤4 vs. >4 weeks). A meta-regression will be performed to investigate and estimate the dose-response adjusted effects using Hedges’ g and a multilevel approach (based on subgroups) via the metafor package and dose-response model using the dosresmeta package in R.^35^

Sensitivity analyses will be carried out by excluding studies with a high risk of bias or using alternate outcome measures. If statistical pooling is not feasible due to clinical, methodological, or statistical heterogeneity, a narrative synthesis will be conducted. This will summarize study characteristics, interventions, and outcomes descriptively, including relevant tables and figures. A funnel plot will be generated in RevMan to assess publication bias when at least 10 studies are available. Egger’s regression test will be used to detect small-study effects. All full-text studies excluded after assessment will be documented, along with reasons for exclusion, and this will be presented in a PRISMA flow diagram and supplementary tables, in accordance with JBI guidance. Statistical procedures will follow guidance from the *JBI Manual for Evidence Synthesis*.^28^

### Assessing certainty in the findings

The overall strength of the body of evidence will be assessed independently by 2 reviewers using the Grading of Recommendations, Assessment, Development, and Evaluation (GRADE) approach.^36^ Any disagreements between reviewers will be resolved with help of a third reviewer. A Summary of Findings will be created using GRADEpro GDT (McMaster University, ON, Canada). The Summary of Findings will present the following information where appropriate: absolute risks for the treatment and control; standardized mean difference; and a ranking of the quality of the evidence based on the risk of bias, directness, heterogeneity, precision, and risk of publication bias of the review results. The following outcomes will be included in the Summary of Findings: balance (eg, BBS, BESTest), postural control, quality of life, functional independence, gait performance, functional mobility, and adverse events (if reported). Each outcome will be rated as high, moderate, low, or very low certainty.

## Author contributions

All the authors declare that they meet the ICMJE criteria for authorship. SP and KR conceived and designed the review protocol, and will act as guarantors of the review. SP, KR, and BJ wrote the manuscript, critically reviewed, and revised it. All the authors read and approved the final version of the manuscript.

## Funding

This project is supported by the SRM College of Physiotherapy, Faculty of Medicine and Health Sciences, SRM Institute of Science and Technology (SRM IST), Kattankulathur, Chennai, India.

## Acknowledgments

We thank the Dean, Professor T. S. Veeragoudhaman and librarian, Mrs. Kalaimani, SRM College of Physiotherapy, for providing the digital resources and for access to subscription-based web databases.

## Appendix I: Search strategy

**Table:**

PubMed		
Searched conducted October 1, 2025		
Search #	Search Query	Records retrieved
**#1**	(”Stroke”[Mesh] OR “Cerebrovascular Accident”[tiab] OR “Brain Ischemia”[Mesh] OR “Intracranial Hemorrhages”[Mesh] OR stroke*[tiab] OR CVA[tiab] OR “Cerebral Infarction”[tiab] OR “Cerebral Hemorrhage”[tiab] OR “Post-stroke”[tiab] OR “Brain Attack”[tiab])	30,001
**#2**	(”Postural Balance”[Mesh] OR “Gait Disorders, Neurologic”[Mesh]OR balance[tiab] OR “postural control”[tiab] OR posture[tiab] OR “postural sway”[tiab]	13,755
	OR “functional balance”[tiab] OR “balance impairment”[tiab] OR “balance training”[tiab]	
	OR “gait performance”[tiab] OR “functional mobility”[tiab] OR postural stability[tiab] OR “balance control”[tiab])	
**#3**	(”Video Games”[Mesh] OR “Virtual Reality Exposure Therapy”[Mesh] OR “Exercise Therapy”[Mesh]	16,737
	OR “Wii Fit”[tiab] OR “Wii Fit Plus”[tiab] OR “Wii Balance Board”[tiab]	
	OR exergam*[tiab] OR “virtual reality rehabilitation”[tiab]	
	OR “game-based rehabilitation”[tiab] OR “active video games”[tiab]	
	OR “video game therapy”[tiab] OR “interactive games”[tiab])	
**#4**	#1 AND #2 AND #3	411
Publication timeline: January 1, 2010 to September 30, 2025		
Article type: randomized controlled trial, controlled clinical trial, clinical trial, clinical study, comparative study, equivalence trial, evaluation study, pragmatic clinical trial		
Age: 18 years or older		
Species: Human		
Sex: Male and female		

## Appendix II: Draft data extraction tool

**Table:**

Section			Subfields																			
**Study identification**			Authors			Year					Country								Journal			
			
**Study design**			RCT			Quasi-experimental					Other experimental study design											
		
**Setting**			Inpatient								Outpatient											
	
**Participants**			Sample size			Age					Stroke type								Functional level			
			
**Intervention**			Wii Fit Plus games used			Session duration					Frequency			Total intervention period					Setting		Delivery mode	
					
**Comparator**			Type of control								Description								Dosage			
		
**Outcomes measured**			Primary outcomes								Secondary outcomes											
	
**Results**			Summary of main findings (per outcome)																			

**Adverse effects**			Any reported side effects or dropouts																			

**Risk of bias notes**			Any appraisal notes relevant to synthesis																			

**Other notes**			Additional observations								Author contact								Registration information			
		
**Section**			**Subfields**																			
**Study identification**			Authors		Year						Country						Journal					
					
**Study design**			RCT		Quasi-experimental						Other experimental study design											
				
**Setting**			Inpatient								Outpatient											
			
**Participants**			Sample size		Age						Stroke type						Functional level					
					
**Intervention**	Wii Fit Plus games used			Session duration		Frequency			Total intervention period			Setting			Delivery mode							
								
**Comparator**	Type of control																			Description		Dosage
						
**Outcomes measured**		Primary outcomes								Secondary outcomes												
			
**Results**		Summary of main findings (per outcome)																				
		
**Adverse effects**		Any reported side effects or dropouts																				
		
**Risk of bias notes**		Any appraisal notes relevant to synthesis																				
		
**Other notes**	Additional observations							Author contact					Registration information									
					
